# Addressing the impacts of COVID-19 on refugee health

**DOI:** 10.1371/journal.pmed.1004050

**Published:** 2022-06-27

**Authors:** Rebecca F. Grais, Emmanuel Baron

**Affiliations:** Epicentre, Paris, France

Refugees are persons who left their country of origin for reasons of real or feared persecution, conflict, violence, or other circumstances that require international protection. Almost all states are party to the 1951 UN Convention on Refugees and its 1967 protocol that require all signatories to provide refugees fundamental rights, respect their physical integrity rights, and adhere to norms of non-refoulement (sending them out of their borders) [1]. Refugees have no political power or representation and are often blamed by governments and members of the public for infectious disease epidemics [2]. In fact, refugees may experience elevated risks of infectious diseases through no fault of their own, and the Coronavirus Disease 2019 (COVID-19) pandemic is no exception.

In an accompanying study in *PLOS Medicine*, Altare and colleagues report results from a retrospective cohort study of COVID-19 epidemiology and heathcare services utilization in Azraq and Zaatari refugee camps in Jordan [3]. More than 5.5 million Syrians have fled the country since the Syrian Civil War broke out in 2011, with most settling in neighboring countries [4]. Syrian refugee settlements (camps) were built following the influx of between 1.4 and 1.8 million Syrians, escaping into Jordan. An estimated 20% live in the settlements, while the majority of refugees live in the host community in Jordan, concentrated primarily in and around Amman. The Zaatari camp, opened in 2012, is the largest, with close to 80,000 residents, and the Azraq camp, opened in 2014, is home to close to 38,000 persons [4].

As there is little information about the effects of the COVID-19 pandemic in humanitarian settings, Altare and colleagues take a closer look at Zaatari and Azraq camps. In September 2020, UNHCR announced the first 2 COVID-19 infections in Azraq settlement. Following the positive tests, the 2 refugees were transferred from the camp to an isolation site. As of September 2020, Jordan had reported around 2,500 COVID-19 cases and 17 deaths. Using a surveillance line list and monthly routine data from UNHCR’s health information system from the 2 camps, Altare and colleagues calculated descriptive statistics of COVID-19 cases and adjusted odds ratios for selected outcomes [3]. These outcomes included COVID-19 confirmation, health service utilization for infectious disease, noncommunicable disease, and maternal and child health. The report of the study by Altare and colleagues is comprehensive and the interpretation well thought out with conclusions pointing to the complexity of situation in the camps. The biases and limitations in the interpretation of the data are well described by the authors, highlighting the difficulties of performing interrupted time series analyses in this setting particularly as COVID-19 is only one of the many factors affecting the population. Despite relatively lower reported COVID in Zaatari and Azarq camps and assurance of adequate health services by Jordan and UNHCR, the population remains in camps, and generalizations to all refugee camps and refugees are to be avoided as well as any false equivalence between refugee health and refugee protection.

Faced with unprecedented challenges, many countries instituted border closures and halted or severely restricted immigration and asylum seeking services due to limited capacity to continue these services. Even countries like Uganda, which hosts over 1.5 million refugees from South Sudan, the Democratic Republic of Congo, and other countries announced the suspension of reception of new refugees and asylum seekers in March 2020 as they were unable to cope with the added burden [5]. However, COVID-19 has also been used as a rationale for migration policies that are not justified by health concerns. Politicians throughout the world have called for tougher actions to limit the mobility of migrants and refugees to counter the threat of the COVID-19, suggesting that they are to blame for spread of the virus, particularly at the onset of the pandemic [6–10]. Altare and colleagues’ findings highlight the likely injustices of these claims.

Lockdowns and movement restrictions during the pandemic have had a devastating toll on refugees, reducing livelihoods and pushing many further into poverty. In December 2020, WHO released the results of a survey, Apart Together, conducted among over 30,000 refugees and migrants from different regions of the world to assess the impact of the COVID-19 pandemic on their mental and physical health and their ability to work and support themselves [11]. Over half of respondents reported greater depression, loneliness, fear, and anxiety. Limited access to information due to language and cultural barriers, coupled with fear of deportation, exacerbated their marginalization. Facilitating refugees rebuilding their lives by focusing on access to education, work opportunities, mitigation, and monitoring of gender and sexual-based violence, and mental health without stigmatization, is even more important during these pandemic times when refugees unnecessarily take the blame.

## References

[1] UNHCR. Frequently asked questions about the 1951 Refugee Convention. [cited 2022 May 10]. Available from: https://www.unhcr.org/uk/news/stories/2001/6/3b4c06578/frequently-asked-questions-1951-refugee-convention.html#_Toc519482140.

[2] Braithwaite A, Frith M, Savun B, Ghosn F. Government Targeting of Refugees in the Midst of Epidemics. Perspect Polit. 2021;1–17. doi: 10.1017/S1537592721001067

[3] Altare C, Kostandova N, OKeeffe J, Hayek H, Fawad M, Khalifa AM, et al. COVID-19 epidemiology and changes in health service utilization in Azraq and Zaatari refugee camps in Jordan: A retrospective cohort study. PLoS Med. 2022;19(5):e1003993. doi: 10.1371/journal.pmed.1003993 35536871 PMC9089859

[4] Syria Refugee Crisis Explained. UNHCR. [cited 2022 May 11]. Available from: https://www.unrefugees.org/news/syria-refugee-crisis-explained.

[5] Uganda borders closed to new refugees, asylum seekers. The Independent March 25, 2020. [cited 2022 May 10]. Available from: https://www.independent.co.ug/uganda-borders-closed-to-new-refugees-asylum-seekers.

[6] Hungary’s Orban blames foreigners, migration for coronavirus spread. France24. [cited 2022 May 10]. Available from: https://www.france24.com/en/20200313-hungary-s-pm-orban-blames-foreign-students-migration-for-coronavirus-spread.

[7] Far right uses coronavirus to scapegoat refugees. New Frame. [cited 2022 May 10]. Available from: https://www.newframe.com/far-right-uses-coronavirus-to-scapegoat-refugees.

[8] Tondo, Lorenzo. Salvini attacks Italy PM over coronavirus and links to rescue ship. The Guardian. [cited 2022 May 10]. Available from: https://www.theguardian.com/world/2020/feb/24/salvini-attacks-italy-pm-over-coronavirus-and-links-to-rescue-ship.

[9] Malaysia migrant raids ’to reduce Covid-19 spread. BBC. [cited 2022 May 11]. Available from: https://www.bbc.com/news/world-asia-52515000.

[10] Lebanon: Refugees at Risk in Covid-19 Response. Human Rights Watch. [cited 2022 May 11]. Available from: https://www.hrw.org/news/2020/04/02/lebanon-refugees-risk-covid-19-response.

[11] World Health Organization. Apart Together survey: Preliminary overview of Refugees and Migrants Self-Reported Impact of COVID-19. 2020. Licence: CC BY-NC-SA 3.0 IGO. Available from: https://www.who.int/publications/i/item/9789240017924.

